# Finite-Size Relaxational Dynamics of a Spike Random Matrix Spherical Model

**DOI:** 10.3390/e25060957

**Published:** 2023-06-20

**Authors:** Pedro H. de Freitas Pimenta, Daniel A. Stariolo

**Affiliations:** 1 Departamento de Física, Universidade Federal Fluminense, Niterói 24210-346, RJ, Brazil; 2 Departamento de Física, National Institute of Science and Technology for Complex Systems, Universidade Federal Fluminense, Campus da Praia Vermelha, Av. Litorânea s/n, Niterói 24210-346, RJ, Brazil

**Keywords:** disordered systems, spike random matrices, eigenvalue statistics, spherical model, Langevin dynamics, non-equilibrium dynamics

## Abstract

We present a thorough numerical analysis of the relaxational dynamics of the Sherrington–Kirkpatrick spherical model with an additive non-disordered perturbation for large but finite sizes *N*. In the thermodynamic limit and at low temperatures, the perturbation is responsible for a phase transition from a spin glass to a ferromagnetic phase. We show that finite-size effects induce the appearance of a distinctive slow regime in the relaxation dynamics, the extension of which depends on the size of the system and also on the strength of the non-disordered perturbation. The long time dynamics are characterized by the two largest eigenvalues of a spike random matrix which defines the model, and particularly by the statistics concerning the gap between them. We characterize the finite-size statistics of the two largest eigenvalues of the spike random matrices in the different regimes, sub-critical, critical, and super-critical, confirming some known results and anticipating others, even in the less studied critical regime. We also numerically characterize the finite-size statistics of the gap, which we hope may encourage analytical work which is lacking. Finally, we compute the finite-size scaling of the long time relaxation of the energy, showing the existence of power laws with exponents that depend on the strength of the non-disordered perturbation in a way that is governed by the finite-size statistics of the gap.

## 1. Introduction

Quenched random interactions are known to be at the origin of complex behavior in many body systems, both in thermodynamics and dynamics [1,2,3]. The understanding of the properties of this kind of systems in the thermodynamic limit has been steadily growing over the last 40 years or so, mainly through research into solutions of mean field, fully connected models, and also from numerical simulations of finite dimensional ones. On the other side, the behavior of systems composed of a large but finite number of degrees of freedom is much less understood. This case is relevant in many applications in many branches of science, e.g., optimization and inference algorithms [4], biological populations [5], and neural networks [6], to cite but a few. Powerful techniques, such as the saddle point method, are not so useful for studying systems far from the thermodynamic limit. There are a few classes of models in which both the thermodynamics and the dynamical behavior can be solved exactly and still show interesting non-trivial properties qualitatively similar to those of more complex systems. Well-known examples include systems in which the degrees of freedom obey a spherical constraint. One of these models with quenched random pairwise interactions, the Spherical Sherrington–Kirkpatrick model (SSK), allows an exact solution of its thermodynamic properties using tools from Random Matrix Theory [7], without the need to use the more involved replica formalism, necessary for the Ising case. The Langevin relaxational dynamics of the SSK model were solved in [8], where it was shown that the long time relaxation is slow, e.g., with the energy density decaying with a power law in time after a quench from a high temperature initial state to a temperature below the spin glass transition temperature. Interesting out-of-equilibrium features of the dynamics, such as the phenomenon of aging, are present in the model and were completely characterized. The solution of both the thermodynamics and the dynamics of the model was possible due to the knowledge of the spectral properties of the random interactions matrix. In the case of the Gaussian Orthogonal Ensemble, the relevant information is in the Wigner semi-circle density of eigenvalues. When the size of the matrix, N×N, is large but finite, the situation changes. The support of the eigenvalue density is not limited anymore, and the probability distribution of the eigenvalues on the soft edge is given by the celebrated Tracy–Widom β distributions [9,10], where β=1,2,4 refer to the orthogonal, unitary, and symplectic ensembles, respectively. Fluctuations of the free energy of the SSK model were studied, e.g., in [11,12,13,14,15]. These fluctuations are governed by the statistics of the largest eigenvalue of the GOE interaction matrix. The finite *N* fluctuations in the Langevin dynamics were studied in [16,17]. In these works, a new algebraic (power law) scaling regime was found and characterized, not present in the N→∞ regime. In the dynamical context, besides the relevance of the largest eigenvalue, which is directly proportional to the ground state energy, the gap between the two largest eigenvalues is also a fundamental quantity to compute the relevant time/size scalings of the long time relaxation.

An interesting related model is the SSK model supplemented with an additive Curie–Weiss term in the Hamiltonian, or equivalently, where the original random interaction matrix is perturbed by a rank one matrix which has the effect of shifting the average value of the random matrix elements from zero to a non zero value. The thermodynamics of this model were also solved in the original work by Kosterlitz et al. [7]. At low enough temperatures, the model presents a phase transition from a spin glass to a ferromagnetic phase at a critical value of the relative strength between the random interactions and the Curie–Weiss one. In the mathematics literature, these kinds of random matrices with finite rank perturbations are called “spike random matrices”. There is a large body of work devoted to the study of the spectral properties of spike random matrices [18,19,20,21,22,23,24,25]. Of special interest to the physics community is the result, originally presented in [19], of a sharp phase transition in the statistics of the largest eigenvalue of particular classes of spike random matrices. Of course, this phase transition has an immediate interpretation in the context of the thermodynamic and also dynamic transitions in the SSK model and related ones. Recently, a renewed interest in the statistical behavior of spike random matrices is manifested in several works focusing on different applications, e.g., overlaps between eigenvectors of correlated spike random matrices [26], analysis of optimal learning rates in non-convex optimization [27], low-rank matrix estimation [28], limits of detection of planted states [29], and ruggedness of complex energy landscapes [30]. From a dynamical perspective, an understanding of spike random matrix models may shed light on problems such as the feasibility of identifying a deterministic signal in a random environment, the reconstruction of hidden patterns in a complex landscape, or the efficiency of search algorithms.

In this work, we perform a numerical study of the statistics of the largest eigenvalues and the gap between the two largest ones in spike random matrices from the GOE ensemble. With the information gained, we then describe the relaxation of the excess energy from the ground state of the spike SSK model following a quench from a high-temperature initial state directly to zero temperature. We show that, as is the case in the standard SSK model, the relaxation shows a new scaling regime, present when the system transitions from the spin glass to the ferromagnetic phases, i.e., a critical scaling regime. In this critical sector, we show that the scaling relaxation behavior is governed by a one-parameter scaling function which depends on the relative strength of the random and Curie–Weiss term and also on the size of the system.

The paper is organized as follows: in Section 2 we introduce the model studied and summarize some known results which will be useful later; in Section 3 we present a numerical study of the statistics of the two largest eigenvalues and the gap of a rank one spike GOE matrix; in Section 4, we present our results on the relaxation dynamics of the model, both in the thermodynamic limit and for large but finite system sizes. Finally, in Section 5, we present a brief discussion of the work and our conclusions.

## 2. The Model

The spherical Sherrington–Kirkpatrick (SSK) model with a Curie–Weiss (CW) perturbation is described by the following Hamiltonian:

(1) $$\begin{array}{cc} H[\vec{S},z] & =H_{SSK}\left(\vec{S}\right)+H_{CW}\left(\vec{S}\right)+\frac{z}{2}\left(\vec{S}{\;}^{2}-N\right)\\ \; & =-\frac{1}{2}\vec{S}{\;}^{T}M\vec{S}+\frac{z}{2}\left(\vec{S}{\;}^{2}-N\right)\\ \; & =-\frac{1}{2\sqrt{N}}\sum_{i\neq j}^{N}J_{ij}\;s_{i}s_{j}-\frac{\theta }{2N}\sum_{i\neq j}^{N}\;s_{i}s_{j}+\frac{z}{2}\left(\sum_{i}^{N}s_{i}^{2}-N\right), \end{array}$$

where *z* is a Lagrange multiplier which enforces the spherical constraint:

(2) $$\vec{S}{\;}^{2}=\sum_{i}^{N}s_{i}^{2}=N.$$

In the previous expressions, the spin variables $s_{i}\in \left[-\sqrt{N},\sqrt{N}\right]$ are described by a *N* component vector $\vec{S}=\left(s_{1},\ldots ,s_{N}\right)$. The coupling constants $J_{ij}$ are chosen form a real and symmetric random matrix from the Gaussian Orthogonal Ensemble (GOE), $J={\left\{J_{ij}\right\}}_{\left(i,j\right)\in {\left[1,N\right]}^{2}}$, with zero mean and variance $J^{2}$. $\theta \in R^{+}$ measures the intensity of the deterministic perturbation. Thus, M is a real symmetric N×N spike random matrix, whose off-diagonal elements are Gaussian distributed with the following mean and variance:

(3) $$p\left(M_{ij}\right)=N\left[\mu =\frac{\theta }{N},\;\sigma^{2}=\frac{J^{2}}{N}\right]\;.$$

The diagonal elements are zero. The Hamiltonian can be rewritten decomposing $\vec{S}$ as a linear combination of the eigenvectors $\left\{{\vec{V}}_{\mu }\right\}$ of the coupling matrix M, with ${\vec{V}}_{\mu }\cdot \vec{V_{\nu }}=\delta_{\mu \nu }$. Therefore, with the following notation $s_{\mu }=\vec{S}\cdot {\vec{V}}_{\mu }$ for the projections of $\vec{S}$ on the eigenvectors of M, the Hamiltonian becomes:

(4) $$H\left[\vec{S},z\right]=-\frac{1}{2}\sum_{\mu =1}^{N}\left(\lambda_{\mu }-z\right)\;s_{\mu }^{2}-\frac{z}{2}N\;,$$

with ${\left\{\lambda_{\mu }\right\}}_{\mu \in [1,N]}$ being the set of *N* eigenvalues of M, with associated eigenvectors $\left\{{\vec{V}}_{\mu }\right\}$. The eigenvalues are organized such that $\mathrm{Max}\left[\lambda_{\mu }\right]=\lambda_{1}>\lambda_{2}>\ldots >\lambda_{N}=\mathrm{Min}\left[\lambda_{\mu }\right]$. In the large *N* limit, the eigenvalue density distribution of M is given by [31]:

(5) $$\rho \left(\lambda \right)=\left\{\begin{array}{cc} \rho_{W}\left(\lambda \right)\;\; & ,\;\;\theta \leq J\\ \rho_{W}\left(\lambda \right)+\frac{1}{N}\delta \left[\lambda -\left(\theta +J^{2}/\theta \right)\right]\;\; & ,\;\;\theta >J \end{array}\right.\;,$$

where $\rho_{W}\left(\lambda \right)$ is the Wigner semicircle law:

(6) $$\rho_{W}\left(\lambda \right)=\left\{\begin{array}{cc} \frac{{\left(4J^{2}-\lambda^{2}\right)}^{1/2}}{2\pi J^{2}}\;\;\; & ,\;\;|\lambda |<2J\\ 0\;\; & ,\;\;|\lambda |>2J \end{array}\right.\;.$$

The result (5) means that, if θ≤J, the spectrum of M is given by the Wigner law, corresponding to the GOE ensemble. Otherwise, if θ>J, the largest eigenvalue $\lambda_{1}$ detaches from the Wigner semicircle, becoming an outlier with a delta peak at $\lambda =\theta +J^{2}\theta^{-1}$.

The following set of Langevin equations governs the overdamped dynamics of the model:

(7) $$\begin{array}{cc} \frac{\partial s_{i}\left(t\right)}{\partial t}\; & =-\delta_{s_{i}}H\left[\vec{S},z\right]+\xi_{i}\left(t\right)\\ \; & =\sum_{j\neq i}^{N}M_{ij}s_{j}\left(t\right)-z\left(t,\left\{s_{\mu }\left(0\right)\right\}\right)s_{i}\left(t\right)+\xi_{i}\left(t\right),\;\;\;\;\forall i\in \left[1,N\right]\;, \end{array}$$

where $\xi_{i}\left(t\right)$ represents a Gaussian white noise with zero mean and variance $\langle \xi_{i}\left(t\right)\xi_{j}\left(t^{\prime }\right)\rangle =2T\delta_{ij}\delta \left(t-t^{\prime }\right)$ and *T* is the temperature of a thermal bath. As in [17], here we are interested in the zero temperature limit of the Langevin equations, which, in the eigenbasis of M, reads:

(8) $$\frac{\partial s_{\mu }\left(t\right)}{\partial t}=\left[\lambda_{\mu }-z\left(t,\left\{s_{\mu }\left(0\right)\right\}\right)\right]s_{\mu }\left(t\right),\;\;\;\;\forall \mu \in \left[1,N\right]\;.$$

At long times, the system must fall in a stable or metastable state of the free energy which, at T=0, reduces to the Hamiltonian $H[\vec{S},z]$. The asymptotic stationary state will depend on the initial conditions $\left\{s_{\mu }\left(0\right)\right\}$, as stated explicitly in (8). By setting $\lim_{t\to \infty }\partial_{t}s_{\mu }\left(t\right)=0$ we obtain the criteria:

(9) $$\delta_{s_{\mu }}H\left[\vec{S},z\right]=-\left(\lambda_{\mu }-z\right)s_{\mu }=0,\;\;\forall \mu \in \left[1,N\right],$$

complemented by the spherical constraint in Equation (2). This system of equations admits the 2N solutions:

(10) $$\vec{S}=\pm \sqrt{N}\;{\vec{V}}_{\mu }\;\;\mathrm{and}\;\;z=\lambda_{\mu }\;\;\forall \mu \in \left[1,N\right].$$

Their stability is determined by the Hessian $\delta_{s_{\mu }}\delta_{s_{\nu }}H\left[\vec{S},z\right]=-\left(\lambda_{\nu }-z\right)\delta_{\mu \nu }$. Taking a given metastable state $\vec{S}=\sqrt{N}{\vec{V}}_{\mu }$, the local landscape has N−μ stable directions, μ−1 unstable directions and a marginal flat one. The energy of each of these configurations is equal to $-\lambda_{\mu }N/2$. Thus, the system should always equilibrate in one of the solutions $\pm \sqrt{N}{\vec{V}}_{1}$, as they are the only stable ones. The ground state energy density is then simply given by $e_{\mathrm{eq}}=-\lambda_{1}/2$.

Our primary interest here is to describe the behavior, at long times, of the excess energy density, $\Delta e\left(t,N\right)=e\left(t,N\right)-e_{\mathrm{eq}}\left(N\right)$, for arbitrary system sizes *N*. To this end, recalling results in [16,17], it can be shown that the time-dependent Lagrange multiplier has a simple relation with the energy density, z(t,N)=−2e(t,N), leading to the exact expression for the excess energy density:

(11) $$\Delta e\left(t,N\right)=\frac{\lambda_{1}}{2}+e\left(t,N\right)=\frac{1}{2}\frac{\sum_{\mu =2}^{N}s_{\mu }^{2}\left(0\right)\left(\lambda_{1}-\lambda_{\mu }\right)\;e^{2(\lambda_{\mu }-\lambda_{1})t}}{s_{1}^{2}\left(0\right)+\sum_{\mu =2}^{N}s_{\mu }^{2}\left(0\right)\;e^{2(\lambda_{\mu }-\lambda_{1})t}}\;.$$

Because of the dependence of the above expression on the relaxation rates $\lambda_{\mu }-\lambda_{1}$, one expects that the late dynamics of the model will be dominated by the gap, $g=\lambda_{1}-\lambda_{2}$, between the two largest eigenvalues of the random matrix M:

(12) $$\Delta e\left(t,N\right)\overset{t\to \infty }{\to }\frac{1}{2}\frac{s_{2}^{2}\left(0\right)}{s_{1}^{2}\left(0\right)}\;g\;e^{-2gt}\;.$$

In the previous expression, there are two sources of fluctuations: the statistics of the gap and the initial conditions. The vector of initial conditions $\vec{S}\left(0\right)=\left\{s_{\mu }\left(0\right)\right\}$ can be written in the basis of eigenvectors of the M matrix in the form $\vec{S}\left(0\right)=\sum_{\nu }c_{\nu }{\vec{V}}_{\nu }$, where the coefficients $c_{\nu }$ satisfy the condition $S^{2}\left(0\right)=\sum_{\nu \eta }c_{\nu }c_{\eta }\;{\vec{V}}_{\nu }\cdot {\vec{V}}_{\eta }=\sum_{\nu }c_{\nu }^{2}=N$. In the present work, we are primarily interested in a flat distribution on the basis of eigenvectors, that is $c_{\nu }=1$ for all ν, which can be associated to thermal equilibrium at a very high temperature. It corresponds to:

(13) $$s_{\mu }\left(0\right)=s_{\mu }^{\mathrm{flat}}\left(0\right)=1\;\;\forall \mu \in \left[1,N\right].$$

The relaxation dynamics of the model depend on the form of the eigenvalue density (5). The effect of the delta contribution is to induce a phase transition when the intensity of the Curie–Weiss term attains the value θ=J in the thermodynamic limit. Although the CW term remains weaker than the random couplings’ intensity, θ<J, the system behaves like the pure SSK model, relaxing towards a disordered ground state $\vec{S}$ with a characteristic slow dynamics, as described in [8,16,17]. At finite temperatures, the thermodynamics corresponds to a spin glass phase, originally described in [7]. On the other hand, if the perturbation is strong enough, θ>J, the largest eigenvalue detaches from the bulk of the spectrum, inducing a fast relaxation towards a ferromagnetic ground state, where all the spin variables $s_{i}$ align in the same direction. For not too high temperatures, exactly at θ=J, the system goes through a continuous phase transition between a disordered spin glass phase and a ferromagnetically ordered one, in the thermodynamic limit [7]. In [13], finite-size fluctuations of the free energy of the model at both sides of the spin glass-ferromagnetic transition were characterized. Here, we are interested in characterizing the finite-size fluctuations of the relaxation dynamics, following a quench from an infinite temperature initial state down to zero temperature for different values of the CW perturbation intensity.

Considering random initial conditions as given by (13), at long times, the behavior of the average excess energy is given by:

(14) $$E\left[\Delta e\left(t,N\right)\right]\overset{t\to \infty }{\to }\frac{1}{2}\;E\left[g\;e^{-2gt}\right]\;.$$

At present, the statistical properties of the gap $g=\lambda_{1}-\lambda_{2}$ are not known. In order to describe its approximate behavior, in the following section, we will pursue a thorough numerical investigation of the statistics of the two largest eigenvalues and the gap of the spike random matrix M, for large but finite system size *N*.

## 3. Statistics of the Two Largest Eigenvalues and the Gap for Finite-Size Spike Matrices

In the limit N→∞, the distribution of eigenvalues is given by (5). When *N* is finite, the border of the spectrum shows finite-size fluctuations. For spike matrices belonging to the complex Wishart ensemble a phase transition was identified in the behavior of $\lambda_{1}$, as the mean value of the elements of the random matrix changes [19,20]. A similar behavior for real Wishart matrices was conjectured in [19] and subsequently confirmed by several approaches (see, e.g., [23,24] and references therein). Extensions for the GOE and other Gaussian ensembles were considered in [24]. Its connection with the thermodynamic phase transition in the SSK model is immediate because the free energy of the model (which reduces to the average Hamiltonian at zero temperature) is proportional to $\lambda_{1}$. Then, when considering finite-size fluctuations at T=0, three regimes are of interest: a sub-critical regime when θ≪J, a critical one when θ∼J, and a super-critical one when θ≫J. The fluctuations of $\lambda_{1}$ in the sub-critical and super-critical regimes have been considered in several works [12,13,21,22,25]. Nevertheless, results on the critical regime are scarce [23,24]. The following results are known: fixing J=1, as long as θ<1, the perturbation has little effect on the behavior of $\lambda_{1}$. In this case its distribution is described by the GOE Tracy–Widom (TW) distribution [10,13,25]:

(15) $$N^{-2\;/\;3}\rho \left[N^{2\;/\;3}\left(\lambda_{1}-E\left[\lambda_{1}\right]\right)\right]⟹TW,\;\theta <1\;.$$

Instead, when θ>1, $\lambda_{1}$ becomes an isolated eigenvalue as it goes away from the support of the semicircle, being freer to fluctuate around the expected value, $E\left[\lambda_{1}\right]=\theta +\theta^{-1}$. In this case, the fluctuation of $\lambda_{1}$ is of order $O(N^{-1/2})$, described by a normal distribution [13,25]:

(16) $$N^{-1\;/\;2}\rho \left[N^{1\;/\;2}\left(\lambda_{1}-E\left[\lambda_{1}\right]\right)\right]⟹N\left[0,\;2(1-\theta^{-2})\right],\;\theta >1\;.$$

**Figure 1:**
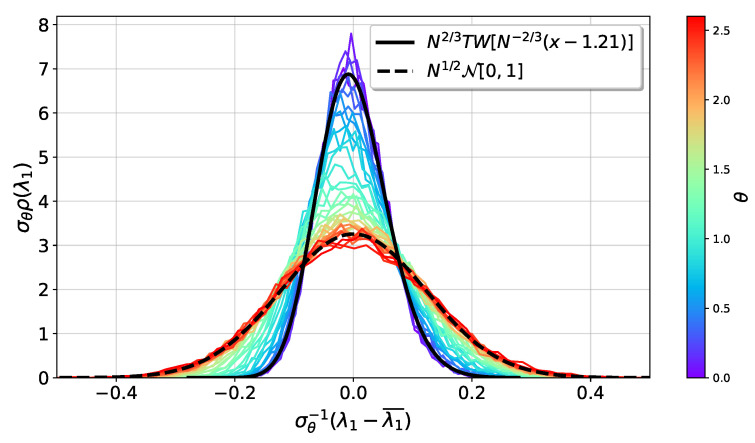
Scaled probability density distributions of an ensemble of $10^{4}$ spike random matrices with N=100. The distributions are centered relative to the ensemble average $\bar{\lambda_{1}}$ and $\sigma_{\theta }$ stands for the predicted standard deviation when θ>1. The centered TW distribution TW (15) and the normal distribution N[0,1] (16) have been scaled similarly to the data.

Figure 1 shows the behavior of the probability density distribution of $\lambda_{1}$, collected from an ensemble of $10^{4}$ spike random matrices of size N=100, for several values of θ, shown in the color scale to the right of the figure. The distributions are centered at zero, $\bar{\lambda_{1}}$ stands for the ensemble average and $\sigma_{\theta }$ is the predicted standard deviation given in (16), applied only in its valid interval θ>1. The Tracy–Widom and the normal distribution, shown in continuous and dashed lines, are properly scaled. The plots of the TW distributions were done by using the publicly available package in https://github.com/yymao/TracyWidom/ (accessed on 10 February 2022). This package uses interpolation tables from [32,33]. In agreement with the results above, for θ≪1, the pdf of the largest eigenvalue is well described by a TW distribution. At the other end, when θ≫1, a normal distribution with the theoretically predicted behavior is observed. A crossover behavior at intermediate values of θ is also observed. This is the critical regime. At present, there are a few results on the behavior of the largest eigenvalue of spike random matrices in the critical regime [23,24], from which we have been able to describe the scaling of the expectation value of $\lambda_{1}$ and $\lambda_{2}$, as will be shown later.

### 3.1. Expectation Value of $\lambda_{1}$

#### 3.1.1. Sub-Critical Regime, θ≪1

**Figure 2:**
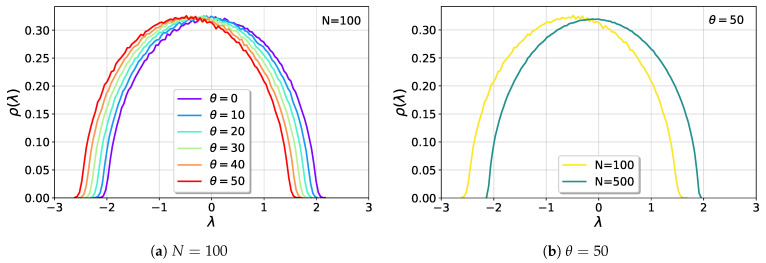
Shift of the semicircle as a consequence of the center of mass conservation described in the text. (**a**) For fixed N=100, the semicircle moves to the left proportionally to the perturbation intensity. (**b**) For fixed θ=50, the shift depends on the size of the matrix: larger sizes *N* suffer smaller shifts.

When θ<1 and for large but finite *N*, $\lambda_{1}$ is expected to behave as $\lambda_{1}=2+\xi \;N^{-2/3}$, where ξ is a random variable described by the GOE TW distribution, with expected value E[ξ]=−1.21 [10,34]. Then, the expected value of $\lambda_{1}$ would behave as $E\left[\lambda_{1}\right]\approx 2-1.21N^{-2/3}$. Nevertheless, the previous result is valid when the diagonal elements of the random matrix are non-null. In the present case, the matrix M is traceless, with all the diagonal elements equal to zero. In Figure 2a, we can see that the semicircle moves approximately linearly to the left as the perturbation intensity increases, while in Figure 2b it is clear that bigger matrix sizes *N* suffer smaller shifts. This is a consequence of the traceless character of the matrix. Upon changing the average value of its elements, θ/N, the eigenvalues will have to rescale their expected values in order to satisfy the condition that they must add up to zero. This is analog to a center of mass conservation of the eigenvalue density.

Then, because for finite *N* the weight of each eigenvalue is 1/N, the expected value of $\lambda_{1}$ should approximately be given by:

(17) $$E\left[\lambda_{1}\right]\approx \left(2-1.21N^{-2/3}\right)\left(1-\frac{1}{N}\right),\;\theta \ll 1.$$

**Figure 3:**
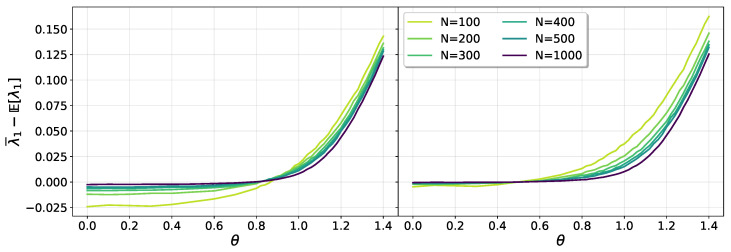
Shift of the numerical average of $\lambda_{1}$ relative to the theoretical prediction (17), for θ<1. The left panel shows results without considering the correction due to the conservation of the center of mass. In the right panel, after inclusion of the correction, the data show a good collapse for growing sizes *N* and sufficiently small values of θ.

Note that because this correction acts equally on every eigenvalue, it will have no effect on the gap $g=\lambda_{1}-\lambda_{2}$, which is the relevant quantity for the long time dynamics. Figure 3 shows the deviation of the numerical average of $\lambda_{1}$ from the theoretical prediction (17). The improvement of the collapse after the inclusion of the center of mass conservation effect is evident in the right panel. It is also possible to note that the collapse breaks down for θ>0.6 for the sizes considered when the system begins to cross over to the critical regime.

#### 3.1.2. Super-Critical Regime, θ≫1

This is the regime in which $\lambda_{1}$ becomes isolated from the bulk. In this case the finite *N* fluctuations are predicted to be Gaussian, given by Equation (16). As in the θ<1 case, the center of mass conservation must be obeyed. We found that it amounts to a shift of the large *N* result $\left(\theta +\theta^{-1}\right)$ by the appropriate weight factor 1/N. Then, for θ≫1, the expected value of $\lambda_{1}$ will be given by:

(18) $$E\left[\lambda_{1}\right]\approx \left(\theta +\theta^{-1}\right)\left(1-\frac{1}{N}\right),\;\theta \gg 1.$$

**Figure 4:**
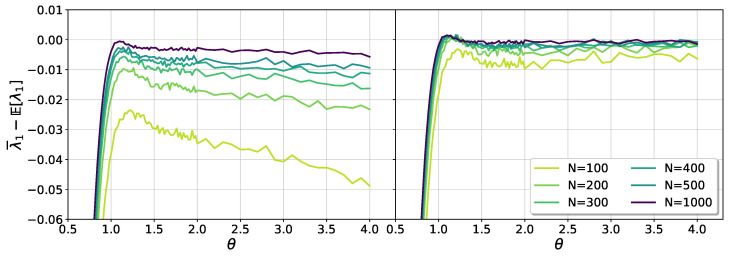
Shift of the numerical average of $\lambda_{1}$ relative to the theoretical prediction (18), for θ>1. The left panel shows results without considering the correction due to the conservation of the center of mass. In the right panel, after inclusion of the correction, the data show a good collapse, improving as *N* grows.

In Figure 4, the effect of the center of mass correction for θ>1 can be appreciated. In this case, besides *N*, there is a dependence on θ, evident in the left panel of the figure. Upon considering the center of mass correction, the result agrees well with Equation (18), when θ≫1.

#### 3.1.3. Critical Regime, θ∼1

**Figure 5:**
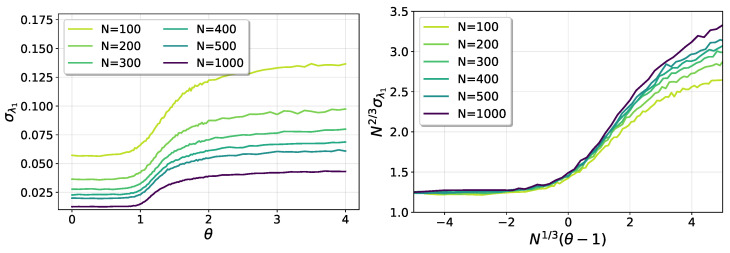
Fluctuations of $\lambda_{1}$ in the critical regime.

In reference [24], the statistics of the largest eigenvalue of spike real Gaussian random matrices in the critical regime are considered. The critical regime is defined for fixed values of the parameter $\omega =N^{1/3}\left(\theta -1\right)\;\in \left(-\infty ,\infty \right]$, and was described originally for the spike complex Wishart ensemble in [19], where the phenomenon of the phase transition in the statistics of the largest eigenvalue was identified. In Theorem 1.5 of [24], it is shown that, in the critical regime, the eigenvalues of spike Gaussian random matrices are given in terms of the eigenvalues of the stochastic Airy operator $H_{\beta ,\omega }$ with suitable boundary conditions. For finite ω, the statistics of the largest eigenvalue $\lambda_{1}$ is described by a “one parameter family of deformations of the Tracy–Widom(β)” distributions, interpolating between the usual values of β=1,2,4. As a consequence, in the critical regime, one expects the $\lambda_{1}$ fluctuations to be approximately described by the TW distribution, but not exactly, with a difference that depends on the value of ω. In Figure 5, we show the (numerical) standard deviation of $\lambda_{1}$ for different system sizes. In the left panel, the raw data are shown as a function of θ. In the right panel, a data collapse is shown, with the scaling variable ω as defined above, assuming fluctuations to scale with $N^{2/3}$, as would be expected for a perfect Tracy–Widom behavior. Although the collapse is good for ω<0 and performs better in the whole interval as the size *N* grows, the quality of the collapse decays as ω grows. According to the results in [24], a continuous change in the exponent, away from 2/3, should be expected.

### 3.2. Expectation Value of $\lambda_{2}$

**Figure 6:**
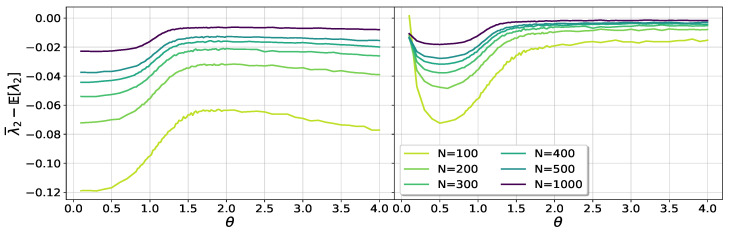
Shift of the numerical average of $\lambda_{2}$ relative to the theoretical prediction (17). The left panel shows results without considering the correction due to the conservation of the center of mass. In the right panel, after inclusion of the correction, the data show a good collapse for growing sizes *N* and sufficiently large values of θ.

As $\lambda_{1}$ jumps outside the semicircle of the Wigner law, it is expected that $\lambda_{2}$ will take its place at the soft edge of the eigenvalue density function. In particular, it is expected that $\lambda_{2}$ will show fluctuations given by the Tracy–Widom distribution. Then, the expectation value of $\lambda_{2}$ should behave as:

(19) $$E\left[\lambda_{2}\right]\approx \left(2-1.21N^{-2/3}\right)\left(1-\frac{1}{N}\right)-\frac{\left(\theta +\theta^{-1}\right)}{N},\;\theta \gg 1,$$

where the first term corresponds to $E[\lambda_{1}]$ in the sub-critical regime, Equation (17), and the second is the correction due to the center of mass conservation when the largest eigenvalue has detached from the bulk. The behavior of the shift of the numerical average ${\bar{\lambda }}_{2}$ from the expectation given by Equation (19) is shown in Figure 6. In the left panel, only the first term on the right-hand side of (19) is shown, while the right panel shows the full expression after taking into account the center of the mass conservation term. A progressive good collapse can be seen in the θ≫1 regime, as *N* grows.

**Figure 7:**
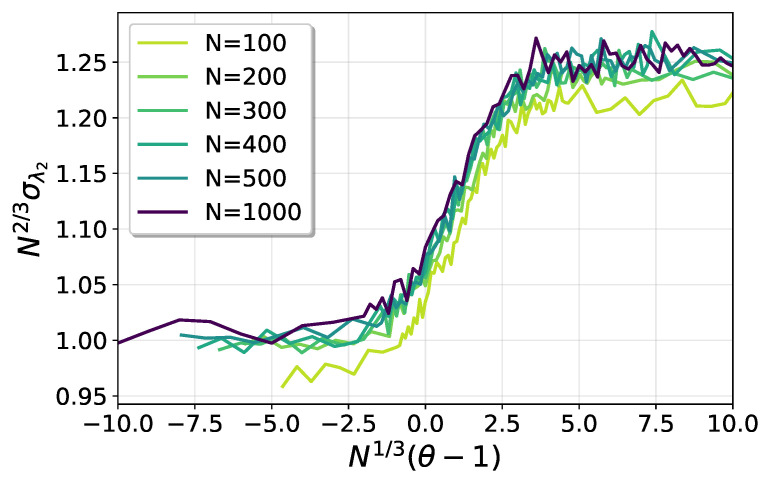
Data collapse of the fluctuations of $\lambda_{2}$.

In Figure 7, we show a data collapse of the fluctuations of $\lambda_{2}$. The collapse is good for the largest sizes, in agreement with theoretical expectations.

### 3.3. Statistics of Small Gaps $g=\lambda_{1}-\lambda_{2}\ll 1$

**Figure 8:**
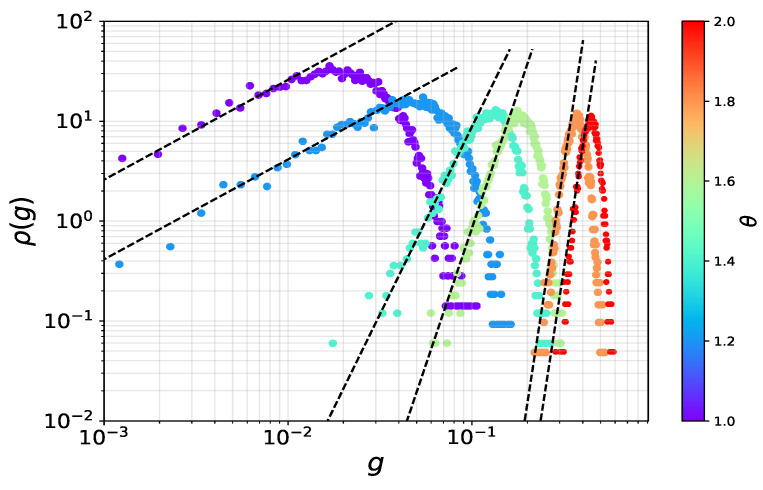
Probability distribution functions of the gap between the two largest eigenvalues for an ensemble of spike random matrices of size N=1000 and different values of the deterministic term intensity θ. The double log scale shows algebraic behavior at small *g*.

The behavior of the gap between the two largest eigenvalues, $g=\lambda_{1}-\lambda_{2}$, will also depend on the regime considered. In the sub-critical regime, the effect of the deterministic perturbation is negligible, and one expects that the statistics of the gap will be governed by the results of reference [35]. In turn, this will lead to power law time/size scalings, as studied in [16,17]. In the super-critical regime, the two largest eigenvalues become approximately independent random variables. From Equations (15) and (16), the Tracy–Widom character of the fluctuations of $\lambda_{2}$, going to zero as $N^{-2/3}$, are sub-leading with respect to the Gaussian fluctuations of $\lambda_{1}$, which decay as $N^{-1/2}$. Then, in the regime θ≫1, one can expect (and we have numerically verified) the fluctuations of small gaps to be Gaussian, like those of $\lambda_{1}$. In turn, this will reflect in exponential time relaxations of observables, such as the energy gap, a typical behavior of ferromagnetic phases. More interesting is the intermediate, critical regime. In this regime, in which $\lambda_{1}$ and $\lambda_{2}$ are strongly correlated, the statistical behavior of the gap is not known. With the aim of describing the long time behavior of the energy gap, we have pursued a numerical characterization of the statistics of the gap in the small gap regime, g≪1, relevant to the long time relaxation dynamics. In Figure 8, the distribution of the gap is shown in double logarithmic scale for an ensemble of random spike matrices of size N=1000 and different values of θ≥1. In all cases, it can be seen that the behavior is algebraic for small *g*.

**Figure 9:**
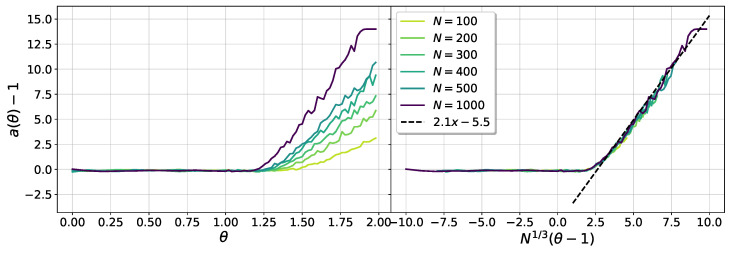
The gap exponent a(θ,N).

Thus, for the small gaps regime, we expect that the pdf of the gap will approximately behave as:

(20) $$f\left(g\right)∼b\left(\theta ,N\right)\;g^{a(\theta ,N)},$$

where a(θ,N) and b(θ,N) are parameters to be determined. We numerically adjusted those parameters to fit the data in the interval $g<E\left[g\right]-\sigma_{g}$. In Figure 9 we show the behavior of the gap exponent with θ, for different system sizes. The left panel shows that a(θ,N) is a constant equal to one in the sub-critical regime, in agreement with the results for the pure SSK model [35]. For θ>1, the numerical analysis suggests a linear behavior, with a slope dependent with *N*. A very good data collapse is obtained as a function of the critical scaling variable $N^{1/3}\left(\theta -1\right)$, as can be seen in the right panel of Figure 9. Then, for θ>1, the gap exponent behaves approximately as:

(21) $$a\left(\theta ,N\right)\approx 1+c_{1}\;N^{1/3}\left(\theta -1\right)-c_{2},$$

where $c_{1}\approx 2.1$ and $c_{2}\approx 5.5$ are fit parameters.

**Figure 10:**
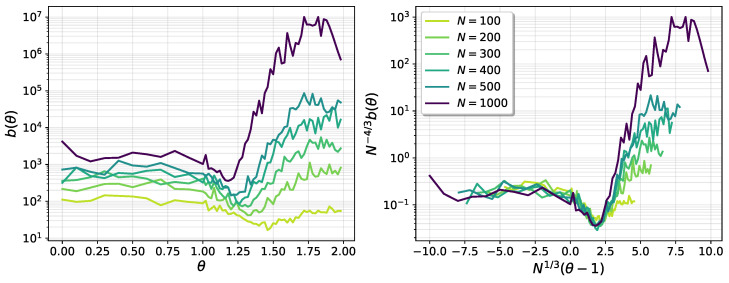
The gap parameter b(θ,N).

Figure 10 shows the behavior of the gap parameter b(θ,N). In the left panel, it can be seen that it presents a minimum at θ>1, which moves towards θ=1 as the system size grows. Then, the critical sector can be identified as the region around the minimum. A good finite-size collapse around the minima is obtained with

(22) $$b\left(\theta ,N\right)=N^{4/3}h\left[N^{1/3}\left(\theta -1\right)\right],$$

where h(x) is an unknown scaling function, as shown in the right panel of the figure.

With these results, it is now possible to compute the finite-size behavior of the time-dependent excess energy, which is the subject of the next section.

## 4. Long Time Decay of the Excess Energy

### 4.1. N→∞ Limit

For random initial conditions given by (13), the Lagrange multiplier is given by [16,17]:

(23) $$z\left(t\right)=\frac{1}{2}\frac{d}{dt}\ln\left[\frac{1}{N}\sum_{\mu =1}^{N}e^{2\lambda_{\mu }t}\right]\overset{N\to \infty }{\to }\frac{1}{2}\frac{d}{dt}\ln\left[\int_{-\infty }^{\infty }d\lambda \;\rho \left(\lambda \right)\;e^{2\lambda t}\right],$$

where the density of eigenvalues, ρ(λ), is given by (5). Performing the integrations, the exact solution is given by:

(24) $$z\left(t\right)=\frac{I_{1}\left(4t\right)}{2t}+e^{2(\theta +\theta^{-1})t},\;\theta >1,$$

where $I_{1}\left(x\right)$ is a modified Bessel function of the first kind. In the long time regime, the above expression has the asymptotic behavior:

(25) $$z\left(t\right)\to \left(\theta +\theta^{-1}\right)+\left[2-\left(\theta +\theta^{-1}\right)\right]\frac{e^{[4-2\left(\theta +\theta^{-1}\right)]t}}{4\sqrt{2\pi }\;t^{3/2}},\;\theta >1.$$

Remembering that the Lagrange multiplier is proportional to the energy density, z(t)=−2e(t) and that, when θ>1, the largest eigenvalue is given by $\lambda_{1}=\theta +\theta^{-1}$, we obtain for the long time behavior of the average excess energy the result:

(26) $$\lim_{N\to \infty }E\left[\Delta e\left(t,\theta \right)\right]=\left\{\begin{array}{cc} \varepsilon (t) & \mathrm{for}\;\theta \leq 1\\ \frac{\left[\left(\theta +\theta^{-1}\right)-2\right]}{8\sqrt{2\pi }}\frac{e^{[4-2\left(\theta +\theta^{-1}\right)]t}}{t^{3/2}} & \mathrm{for}\;\theta >1, \end{array}\right.$$

where ε(t)=3/8t is the known result for the pure SSK model [8]. We note that, as expected, in the θ>1 ferromagnetic regime the asymptotic relaxation, when $t\gg {\left[4-2\left(\theta +\theta^{-1}\right)\right]}^{-1}$, is exponential, faster than in the spin glass phase of the model.

### 4.2. Finite System Size

With the results obtained for the pdf of the gap in the small gap regime for finite *N*, we can compute the late time behavior of the average excess energy, as given by Equation (14):

(27) $$E\left[\Delta e\left(t,\theta ,N\right)\right]=\int_{0}^{\infty }g\;e^{-2gt}\;f\left(g\right)\;dg.$$

**Figure 11:**
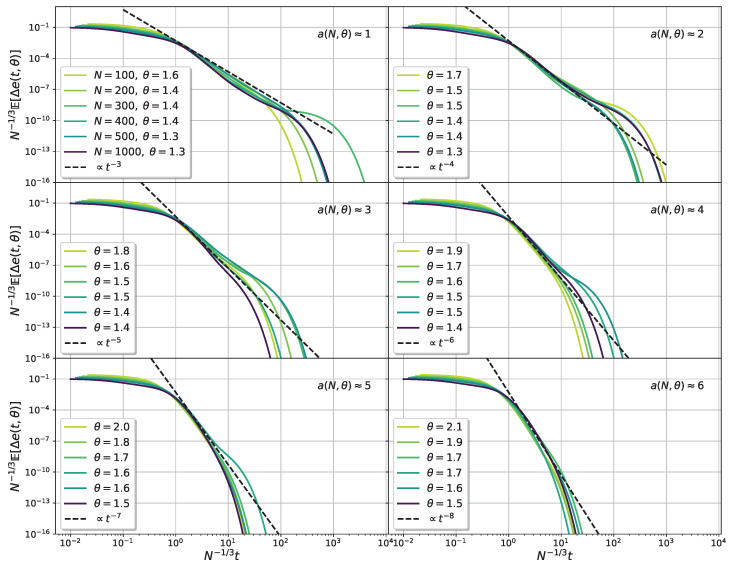
Data collapse of the time decay of the average excess energy for the spike SSK model, according to Equation (30), for different values of the parameter a(θ,N). The slope of the algebraic regime is governed by the one-parameter scaling function $f_{a}\left(x\right)$.

The exact behavior of f(g) for large gaps is not known. Nevertheless, an inspection of the numerical behavior of f(g) (see, e.g., Figure 8) shows that there is a scale $r_{N}$ such that for $g\gg r_{N}$ the gap distribution density shows a fast monotonic decrease towards zero. Then we can write:

(28) $$\begin{array}{ccc} E[\Delta e(t,\theta ,N)] & \approx & b\left(\theta ,N\right)\int_{0}^{r_{N}}dg\;g\;e^{-2gt}\;g^{a(\theta ,N)}+\int_{r_{N}}^{\infty }dg\;g\;e^{-2gt}\;h\left(g/r_{N}\right)\\ & = & b\left(\theta ,N\right)\;t^{-(2+a)}\int_{0}^{r_{N}t}dx\;x^{1+a}\;e^{-2x}+r_{N}^{2}\;\int_{1}^{\infty }dx\;x\;e^{-2(r_{N}t)x}\;h\left(x\right), \end{array}$$

where h(x) is a monotonic decreasing function of its argument. At long times, $r_{N}t\gg 1$, the second integral becomes negligible, and then:

(29) $$E\left[\Delta e\left(t,\theta ,N\right)\right]\approx b\left(\theta ,N\right)\;t^{-(2+a)}\left[2^{-(2+a)}\left(\Gamma \left(2+a\right)-\Gamma \left(2+a,2r_{N}t\right)\right)\right],$$

with the limit $\lim_{r_{N}t\to \infty }\Gamma \left(2+a,2r_{N}t\right)=1$. From (22), (29) and the behavior observed in the numerical results of Figure 11, the long time behavior of the average excess energy, in the critical regime, can be described as follows:

(30) $$E\left[\Delta e\left(t,\theta ,N\right)\right]∼\left\{\begin{array}{cc} \upsilon (t) & \mathrm{for}\;t<N^{1\;/\;3}\\ N^{1\;/\;3}\;f_{a}\left(tN^{-1\;/\;3}\right) & \mathrm{for}\;t>N^{1\;/\;3} \end{array}\right.$$

The function υ(t) represents the time relaxation of the excess energy in the thermodynamic limit, given by (26) when θ>1 and $f_{a}\left(x\right)=c\;/\;x^{2+a}$ is a one-parameter scaling function, with *a* given by (21) and *c* a constant. It is to be noted the difference between the previous results and those for the pure SSK model [16,17]. When θ>1 and $t<N^{1/3}$, the relaxation is exponential, given by υ(t) instead of the power law in the pure case. Additionally, the scaling exponent 1/3 of the algebraic regime differs from the 2/3 of the pure SSK model. In the limit a→1, the expression leads to the expected behavior for the pure model $E\left[\Delta e\right]∼N^{4/3}/t^{3}$ [16,17].

In Figure 11, we can see data collapses of the average excess energy of the spike SSK model, for different fixed values of the parameter a(θ,N). They show good agreement with the previous results. Similarly to the pure SSK model, there is an algebraic scaling regime not present in the N→∞ limit. In this case, the exponent 2+a(θ,N) depends on both the spike intensity θ and the size of the system *N*, according to (21). In the figures, particular combinations of θ and *N* were chosen in order to keep the value of a(θ,N) approximately constant. As the exponent grows, so does the slope of the power law. The relaxation becomes faster, but a power law regime can be identified by values of the parameter *a* as large as a=6, as shown in the figure.

## 5. Discussion and Conclusions

In the first part of this work, we have presented a numerical study of the statistics of the two largest eigenvalues and the gap for random matrices from the Gaussian Orthogonal Ensemble perturbed by a deterministic rank one matrix. The largest eigenvalue of such spike random matrices is known to go through a phase transition as the intensity of the deterministic perturbation attains a critical value [19,24]. The statistics in the sub-critical and super-critical regimes are well described in the literature, while results for the critical regime are scarce [23,24]. Our numerical analysis of the average values of the two largest eigenvalues confirmed the analytical results from the literature, after the inclusion of additional size effects due to the traceless character of the matrices considered in this work; these results are of interest in physics models. In the critical regime, we showed results on the fluctuations of $\lambda_{1}$ and $\lambda_{2}$ in good agreement with available theoretical predictions on the existence of a critical scaling regime where the fluctuations are described by a one-parameter family of scaling functions, which can be seen as continuous deformations of the Tracy–Widom distribution [23,24]. Although our results are compatible with that conclusions, more work is needed to extend and clarify the interpretation of the results from the mathematical literature in the physical models context.

For the statistics of the gap, at present, there are no known analytical results. Then, we pursued a numerical characterization of the small gap regime of the probability density function, which is the relevant regime for the long time behavior of physical observables. We show evidence that the pdf of the gap has a power law behavior for small gaps. The exponent of the power law depends on both the intensity of the deterministic perturbation θ and the system size *N*, in the form which defines the critical sector of the model, $a\left(\theta ,N\right)∼N^{1/3}\left(\theta -1\right)$, when θ>1. After the characterization of the pdf of the gap, we described the long time decay of the average excess energy of the Spherical Sherrington–Kirkpatrick model with a Curie–Weiss perturbation term. We first obtained the analytical result in the large *N* limit, showing that, as expected, the relaxation is exponential for θ>1. We then considered the large but finite *N* behavior. The most interesting and new regime to describe is near the phase transition between the spin glass and ferromagnetic phases. In this critical regime, using the results obtained for the gap pdf, we showed the existence of a sector with power law relaxation as a function of the scaling variable $tN^{-1/3}$.

The results for the gap pdf and the excess energy relaxation are our main new results, not previously reported in the literature. Being mainly of a numerical character, we expect that they will motivate us to pursue analytical approaches to the computation of the gap probability distribution function, which has been shown to be a relevant random variable to describe the late time dynamics of spherical models with pairwise interactions.
